# Investigating the Biosynthesis of Natural Products from Marine Proteobacteria: A Survey of Molecules and Strategies

**DOI:** 10.3390/md15080235

**Published:** 2017-08-01

**Authors:** Marshall L. Timmermans, Yagya P. Paudel, Avena C. Ross

**Affiliations:** Department of Chemistry, Queen’s University, Kingston, ON K7L 3N6, Canada; 15mlt4@queensu.ca (M.L.T.); y.paudel@queensu.ca (Y.P.P.)

**Keywords:** biosynthesis, proteobacteria, natural products, genomics

## Abstract

The phylum proteobacteria contains a wide array of Gram-negative marine bacteria. With recent advances in genomic sequencing, genome analysis, and analytical chemistry techniques, a whole host of information is being revealed about the primary and secondary metabolism of marine proteobacteria. This has led to the discovery of a growing number of medically relevant natural products, including novel leads for the treatment of multidrug-resistant *Staphylococcus aureus* (MRSA) and cancer. Of equal interest, marine proteobacteria produce natural products whose structure and biosynthetic mechanisms differ from those of their terrestrial and actinobacterial counterparts. Notable features of secondary metabolites produced by marine proteobacteria include halogenation, sulfur-containing heterocycles, non-ribosomal peptides, and polyketides with unusual biosynthetic logic. As advances are made in the technology associated with functional genomics, such as computational sequence analysis, targeted DNA manipulation, and heterologous expression, it has become easier to probe the mechanisms for natural product biosynthesis. This review will focus on genomics driven approaches to understanding the biosynthetic mechanisms for natural products produced by marine proteobacteria.

## 1. Introduction

Natural products have long served as the inspiration for a great number of medicinal compounds. Throughout history, from acetylsalicylic acid to Taxol, many of the world’s most effective medicines have been derived from the products of organisms as diverse as plants, fungi, bacteria, and even mammals [1]. The increasing threat of antibiotic resistant pathogens such as *Staphylococcus aureus* and *Mycobacterium tuberculosis* [2], the lack of effective treatments for antibiotic resistant Gram- negative pathogens like *Pseudomoas aeruginosa* [3] and *Escherichia coli* O104:H4 [4], as well as the lack of effective treatments for major diseases such as Cystic Fibrosis [5] and various cancers, creates an impetus for discovery and development of novel therapeutic agents. One approach, once commonly used by industry but now largely driven by academic labs, involves the discovery of biologically active novel natural products [6]. A number of natural products from the marine environment have been approved as drugs including ziconotide, a peptide analgesic derived from the toxin of the marine cone snail *Conus magus*, and the anticancer agent trabectedin derived from the marine tunicate *Ecteinascidia turbinate* [7]. While the marine environment has been a fruitful source of novel molecular scaffolds and bioactive compounds, the industry is reticent to commit resources to natural product discovery in large part due to inherent problems with activity guided fractionation: frequently considerable time and resources are devoted to characterizing a biological extract before it is discovered that the bioactive molecule is already known [8].

One method that may help to avoid natural product rediscovery is genomic analysis [9]. As the technologies associated with DNA sequencing, synthesis, and manipulation become more sophisticated and affordable, large databases of sequence information for many organisms have become available and researchers can more readily manipulate and interrogate the function of genetic elements. It is now possible to sequence an organism’s genome, search for sequences that are similar to known biosynthetic elements, and propose a hypothetical biosynthetic pathway and structure for the encoded molecule. As the number of proven links between genetic elements and natural product structures increases, the accuracy and predictive power of this genome-guided approach to natural product discovery will likewise increase.

Understanding how and why natural products are biosynthesized is equally relevant to researchers studying these molecules. Because many natural product molecules have been discovered from bacteria, particularly from terrestrial actinobacteria such as *Streptomyces* [10], our understanding of how and why natural products are synthesized is based on the mechanisms employed by these organisms. For example, methods for the genomic identification and analysis of Non-Ribosomal Peptide Synthetase (NRPS) and Polyketide Synthase (PKS) biosynthetic pathways are predominantly based on pathways found in actinobacteria. NRPS and PKS biosynthetic pathways function through the activity of large, multidomain “megasynthase” enzymes whose structure and orientation within a multiprotein complex determine the structure of their small-molecule product (Reviews on the general mechanism of typical NRPS and PKS biosynthetic pathways can be found in [11,12,13]). Amino acids are incorporated into a non-ribosomal peptide by “modules” composed of a minimum of three domains. Each amino acid is selected by an adenylation (A) domain and then covalently tethered to the phosphopantetheine cofactor of a peptidyl carrier protein (PCP) domain, the amino acid is then condensed with a second PCP-bound amino acid on an adjacent module through the activity of a condensation (C) domain. Peptides are frequently modified during elongation through the activity of tailoring domains such as methyltransferase, cyclization, and epimerization domains. The growing peptide is passed from one PCP domain to the next along the megasynthase in a linear manner, such that the structure of a peptide can be predicted based on the arrangement of modules within the NRPS biosynthetic pathway. PKS pathways are analogous to NRPS pathways; beta keto acid building blocks are selected by acyltransferase (AT) domains, tethered to acyl carrier protein (ACP) domains, and added to the growing molecules by a Claisen condensation catalyzed by ketosynthase (KS) domains. As marine bacteria are investigated more thoroughly as alternative sources of novel natural products [14,15,16,17,18], the molecular scaffolds and biosynthetic mechanisms observed for marine NRPS and PKS pathways appear to deviate significantly from those found in terrestrial bacteria [19,20]. It appears that marine bacteria may display a greater diversity/frequency of non-canonical biosynthetic mechanisms, particularly through non-linear or iterative NRPS activity when compared to terrestrial microorganisms, additionally a greater number of halogenated natural products have been identified from marine sources likely due to the higher concentration of halides in seawater compared to soil [21,22,23].

**Figure 1:**
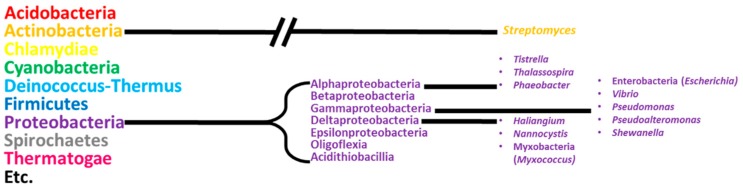
Overview of the relationship between marine proteobacteria and other major phyla of Eubacteria. The sub-phyla of proteobacteria and genera of marine proteobacteria discussed in this review are shown on the right, with non-marine proteobacterial genera used as hosts in heterologous expression experiments shown in brackets.

The phylum proteobacteria are a source of novel natural product leads amongst marine bacteria and have received considerably less attention than actinobacteria, their relationship to other bacterial phyla is shown in Figure 1. Proteobacteria is a diverse class of Gram-negative bacteria that encompasses a number of medically important species, such as the genera *Neisseria, Yersinia,* and *Bortadella*, as well as technologically important genera such as *Agrobacterium, Rhizobium* and *Escherichia* [24].

Proteobacteria also include a number of petroleum-degrading bacteria such as *Alcanivorax, Marinobacter,* and *Pseudoalteromonas* [25]. In addition to pathogenic and soil-associated bacteria, many species of proteobacteria inhabit marine or brackish environments, and often form symbiotic associations with coral, sponges, and molluscs. The number of interesting natural products identified from marine proteobacteria has increased significantly over the years, with novel structures and biosynthetic mechanisms being proposed. This review will focus on natural products produced by marine proteobacteria for which there is significant evidence linking genetic elements to natural product synthesis, as well as the biosynthetic mechanisms proposed based on functional genomic experiments. It will also highlight the variety of genetic and biochemical experiments that have enabled the characterization of these novel natural product biosynthetic pathways.

## 2. Alphaproteobacteria

Alphaproteobacteria is composed of many marine and terrestrial genera with a diversity of lifestyles and metabolisms. These include a number of plant symbionts such as *Rhizobium,* livestock pathogens such as members of the genus *Brucella,* and potentially the bacterial endosymbiont that gave rise to mitochondria [25,26,27]. The alphaproteobacteria contain the genus *Pelagibacter,* one of the most abundant marine microbial organisms [28].

**Figure 2:**
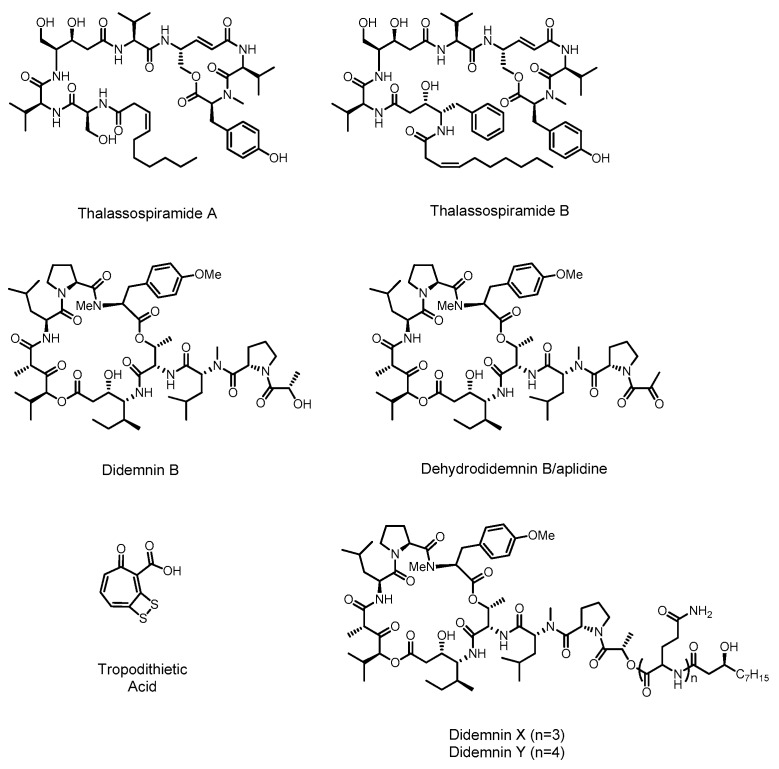
Alphaproteobacterial secondary metabolites with predicted and known biosynthetic origins.

Few alphaproteobacterial secondary metabolites have been isolated and genome analysis with prediction software such as antiSMASH [29,30,31,32] has likewise revealed modest numbers of predicted biosynthetic pathways. Correspondingly only a small number of biosynthetic pathways have been linked to their products in marine alphaproteobacteria, specifically those of didemnin, thalassospiramide, and tropodithietic acid (Figure 2). Nevertheless, the biosynthetic information that we have about this family is intriguing as these bacteria use unusual mechanisms to synthesize non-ribosomal peptides and polyketides.

### 2.1. Didemnin

The didemnin family of molecules are depsipeptides produced by a hydrid NRPS/PKS assembly line in the genus of alphaproteobacteria known as *Tistrella* [33,34,35]. These molecules have a range of biological effects that make them important drug leads. Didemnin B was the first marine natural product to enter clinical trials, being tested as an anti-cancer, anti-viral and immunosuppressive agent. However, toxicity problems discovered in phase II trials stopped further development of this molecule [36,37,38]. Another member of the same molecular family known as dehydrodidemnin B, or aplidine, has continued in trials, and in 2003 was granted orphan drug status for the treatment of acute lymphoblastic leukemia. Aplidine is also currently being investigated for treatment of other cancers at the phase I, II and III levels [36].

**Scheme 1:**
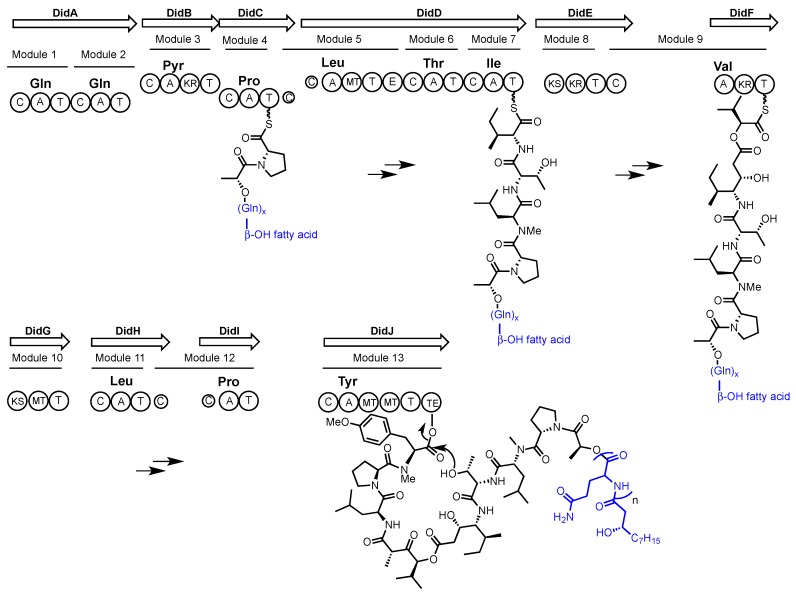
Proposed assembly-line biosynthesis of didemnin. Biosynthetic domains are denoted as: C = condensation, A = adenylation, T = Thiolation, KR = ketoreductase, MT = methyltransferase, E = epimerization, KS = ketosynthase, TE = thioesterase. The amino acid selectivity of each adenylation domain is written above the domain.

Didemnin B and aplidine (see Figure 2) were first identified from extracts of the marine ascidians (sea squirts) *Trididemnum solidum* and *Aplidium albicans* respectively [35,39]. Although postulated as molecules of bacterial origin it wasn’t until 2011 that a marine alphaproteobacterium, *Tistrella mobilis*, was confirmed as a source of didemnin B [33]. Yu et al. subsequently reported the *T. mobilis* genome sequence and the putative biosynthetic gene cluster encoding didemnin B [34]. As previously predicted, a hybrid NRPS/PKS pathway was identified. Intriguingly, no acyltransferase (AT) domain is present in the pathway, and the only other AT encoded in the genome is related to fatty acid synthesis. Although uncommon, the recruitment of AT activity from primary metabolism has been seen previously in the biosynthesis of another NRP/PK hybrid molecule FK228 [40]. Inspection of the modules and domains of the NRPS/PKS system in *T. mobilis* predicts the incorporation of the unusual ketide-extended amino acid residues isostatine (Ist) and hydroxyisovaleryl propionic acid (Hip) in the didemnins. A closer inspection of the didemnin gene cluster with an assumption of collinear biosynthetic logic suggested that the initial product might not be didemnin B but rather an elongated didemnin molecule (see Scheme 1). This is because the gene cluster contains an additional gene (*didA*) encoding two additional modules that are predicted to incorporate two glutamine residues and *N*-terminal acylation with a fatty acid. MALDI-TOF MS was used to search for elongated didemnin molecules in bacterial extracts, resulting in the identification of previously characterized didemnin X and Y [41]. Although these molecules have the predicted *N*-acylated *N*-terminus, they contain more than the two glutamine residues that would be predicted by collinear biosynthesis. This suggested that these molecules might arise from a ‘stuttering’ mechanism where the growing non-ribosomal peptide revisits one or both DidA modules during elongation.

The authors proposed the products of the didemnin NRPS/PKS are didemnin X and Y, which could function like pro-drugs. As lipopeptides these molecules would be more easily exported from the cell, whereupon enzymatic hydrolysis would yield didemnin B. MALDI imaging MS was used to probe this hypothesis. Colonies of *T. mobilis* were imaged over a time-course; revealing that didemnins X and Y were excreted from the cell during days 1–3 of growth, and then from day 4 didemnins X and Y were no longer evident and didemnin B accumulated.

Purified didemnins X and Y were subsequently incubated with a filtered protein extract derived from a *T. mobilis* culture. Once again hydrolysis of didemnins X and Y to form didemnin B was observed. This reaction did not occur in the negative control without bacteria. The authors report that the corresponding transport and lytic enzymes are not known, although they identified several possible candidates encoded near the gene cluster. A similar pro-drug approach is seen in the biosynthesis of the antibiotic xenocoumacin, in which an NRPS derived pre-xenocoumacin is cleaved by a peptidase (XcnG) to generate the mature xenocoumacin that is simultaneously exported from the cell by a partner ABC transporter [42].

To date no functional experiments have been undertaken to further probe the connection between didemnin B and its gene cluster. With the on going clinical relevance of aplidine [43], development of a high-yielding bacterial expression system for this molecule would be extremely useful.

### 2.2. Thalassospiramide

The thalassospiramides are a large family of hybrid molecules produced by an NRPS/PKS assembly line [44]. These molecules are recognized as very potent human calpain 1 protease inhibitors [45] and display neuroprotective properties that could be applied to the treatment of conditions such as Alzheimer’s disease [46]. Thalassospiramides A and B (see Figure 2) were first identified in 2007 by Fenical and co-workers from the alphaproteobacterium *Thalassospira* sp. CNJ-328 [47]. Subsequently in 2013, two further publications expanded the thalassospiramide family to 17 members, and discovered production in another alphaproteobacterial genus, *Tistrella* [44,48]. A recent report by Ross, Qian and co-workers investigated thalassospiramide production by the bacterial family *Rhodospirillaceae*, of which both *Thalassospira* and *Tistrella* are members, identifying 21 additional molecules and a third producing bacterial genus, *Oceanibaculum* [46].

Thirty seven members of the thalassospiramide family contain a conserved *C*-terminal macrocycle that is important for their protease inhibitory activity [45]. In contrast, extensive structural variation is found in the *N*-terminal portion of the molecules. Thalassospiramides have several different chain lengths, with multiple amino acid substitutions (including statine amino acids), several *N*-methylation patterns, and variable *N*-terminal fatty acids. Such diversity can be viewed as a biosynthetic combinatorial library. What is truly remarkable about this group of molecules is the fact that they are almost all produced by a single, biosynthetically flexible, NRPS/PKS system.

**Scheme 2:**
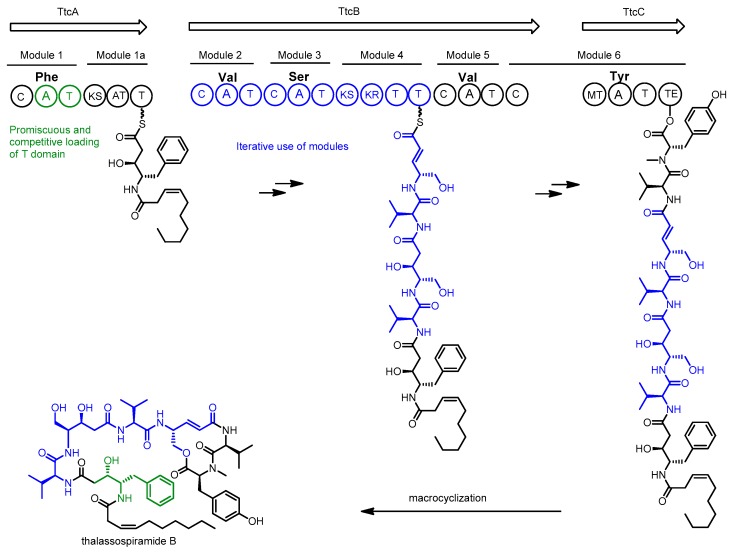
Proposed biosynthesis of thalassospiramides by *Thalassospira* sp. CNJ-328. Biosynthetic domains are denoted as: C = condensation, A = adenylation, T = Thiolation, KR = ketoreductase, MT = methyltransferase, KS = ketosynthase, TE = thioesterase. The amino acid selectivity of each adenylation domain is written above the domain.

Sequencing of the *Thalassospira* sp. CNJ-328 genome in 2013 revealed a single NRPS/PKS gene cluster (*ttc*) candidate for the production of thalassospiramides [44]. Inspection of the encoded genes revealed 7 biosynthetic modules, which would suggest production of only the smallest of the thalassospiramides if using the assumption of collinear biosynthesis. Because most members of the thalassospiramide family possess more than 7 units, the authors proposed an unprecedented multi-modular iteration of peptide synthesis to account for the production of longer molecules (see Scheme 2).

In this model, the growing peptide chain would be translocated back along the assembly line, allowing it to be extended by modules 2, 3 and 4 several times. Amino acid diversity at the *N*-terminal position appears to arise from more than simple adenylation domain promiscuity. An in-vitro assay of the first C-A-T module in the pathway found only phenylalanine and tyrosine could be loaded onto the carrier protein by the adenylation domain. The authors propose the incorporation of serine and valine at the *N*-terminal position of the peptide may arise from cis-acting adenylation domains from elsewhere within the same pathway, which compete with the module 1 adenylation domain to load the module 1 carrier domain. This idea is supported by inspection of the proposed pathway from *Tistrella* where there is a truncated, and presumably non-functional, A domain in the first module that cannot be responsible for loading the carrier protein [49]. Several new variants of the thalassospiramide biosynthetic gene cluster were discovered in 2016 upon inspection of publicly available genomes and the additional sequencing of twenty-six *Thalassospira* genomes and an *Oceanibaculum pacificum* genome as part of a comprehensive investigation of *Rhodospirillaceae* [46].

Additional non-canonical biosynthetic mechanisms were invoked to explain structural variations, including module skipping, stuttering, and multi-module iteration of modules 1 to 4. Interestingly, although every sequenced bacterium possessed a thalassospiramide biosynthetic gene cluster, several bacteria did not have detectable molecule production. This included a fourth genus of α-proteobacteria known as *Fodinicurvata* [50]. Based upon comparative genomics experiments the authors propose a lack of molecule production by these strains may be tied to missing genes that code for enzymes involved in amino acid metabolism, molecule efflux, and signal transduction processes outside the primary cluster.

Although no gene inactivation or heterologous expression experiments have been used to link the thalassospiramides directly to their proposed biosynthetic gene clusters, the presence of some variant of the proposed gene cluster in every strain known to produce the molecules (22 strains across 3 genera) lends considerable weight to the proposed role of the cluster. Furthermore, in the case of the *Thalassospira* strains, there is only a single NRPS gene cluster encoded in each genome.

It is worthy of note that both the thalassospiramide and didemnin pathways appear to utilize trans AT domains from primary metabolism and display module stuttering. Because few biosynthetic pathways from marine alphaproteobacteria have been studied, it remains to be seen if this will become a common theme for alphaproteobacteria or if these two examples are anomalous.

### 2.3. Tropodithietic Acid

Tropodithietic acid (TDA) is a disulfide containing molecule produced by marine alphaproteobacteria of the genus *Phaeobacter* and shows antibiotic activity against a range of Gram-positive and Gram-negative bacteria [51]. TDA has been investigated as a biocontrol agent for preventing vibriosis in aquaculture [52] and has potential anticancer applications due to its ability to disrupt the proton-motive force of a wide variety of cell membranes [53]. *Phaeobacter* and other members of the *Roseobacter* clade are notable for their symbiosis with marine algae, where the production of antibiotic compounds by *Roseobacter* appears to limit levels of marine *Vibrio* species that compete with the algae. In return, *Roseobacter* feeds on lignins produced by the algae [54]. *Phaeobacter* also play a significant role in the marine sulfur cycle by converting the algal metabolite dimethylsulfonioproprionate to dimethyl sulfide [55].

**Scheme 3:**
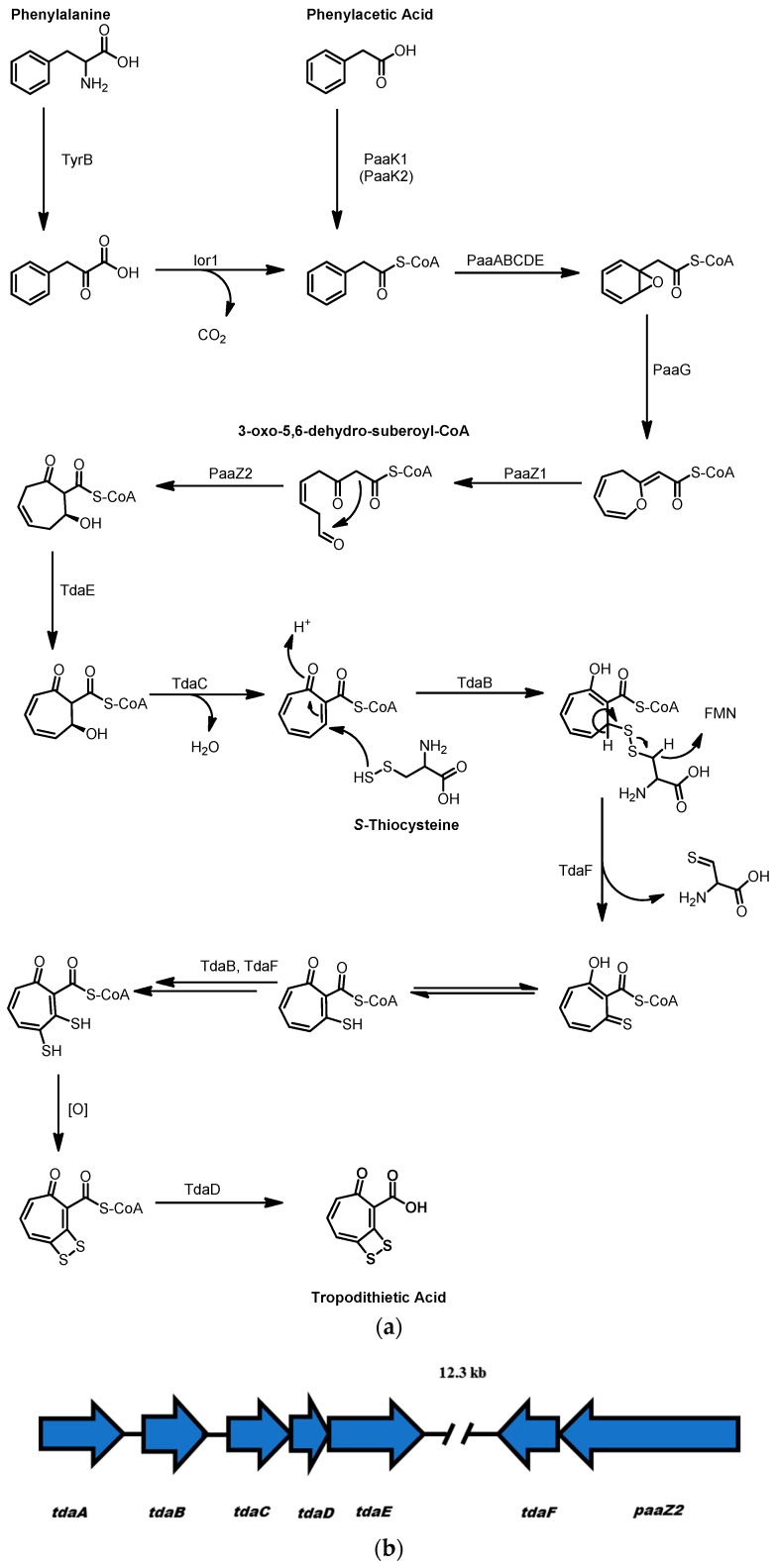
(**a**) Proposed biosynthesis of tropodiethietic Acid (TDA); (**b**) The structure of the tropodithietic acid biosynthetic gene cluster, the genes *tdaA–E* are ~12 kb distant from *tdaF* and *paaZ2.*

TDA has an unusual structure consisting of a seven-membered aromatic tropone ring and a four-membered dithiet ring containing a disulfide bond. The tropone ring is derived from the uncommon metabolite 3-oxo-5,6-dehydro-suberoyl-CoA, a linear 8-carbon beta-gamma unsaturated diketone whose terminal ketone participates in an intramolecular Knoevenagle condensation reaction to form the 7-membered ring (see Scheme 3) [56]. Oxidation by the acyl-CoA dehydrogenase TdaE and elimination of water, catalyzed by the dehydratase TdaC, yields the tropone system. The formation of the dithiet moiety could arise through TdaB catalyzed transfer of sulfur from *S*-thiocysteine to the vinylogous Michael acceptor in the tropone ring to form a tropone-cysteine disulfide adduct. The flavoprotein TdaF using flavin mononucleotide (FMN) as the cofactor could then oxidize the disulfide to a tropone thialdehyde [57]. Following tautomerization of the thioaldehyde, a second round of thiolation by TdaB, oxidation by TdaF, and tautomerization would introduce the second, adjacent thiol, which is then proposed to spontaneously oxidize to form the dithiet moiety. Finally, cleavage of the tropone-dithiet from CoA by the thioesterase TdaD produces tropodithietic acid.

Intriguingly the 3-oxo-5,6-dehydro-suberoyl-CoA that forms the tropone ring is derived from a phenylalanine/phenylacetic acid catabolic pathway [58]. Random transposon mutagenesis of the TDA producer *Phaeobacter galaeciensis* showed that PaaK1 and PaaK2 function as CoA ligases that convert phenylacetic acid to phenylacetyl-CoA. However, double mutants where in both paaK1 and paaK2 were inactivated still produced TDA through an alternate method of phenylacetate formation via an indole oxidoreductase encoded by *ior1* that accepted phenylpyruvate as a substrate to form phenylacetyl-CoA. [58] The function of *ior1* was further elucidated by feeding a strain of *P. galaeciensis* deficient in PaaC, part of the multicomponent oxygenase PaaABCDE, with [1-^13^C] phenylalanine, [2-^13^C] phenylalanine, and [3-^13^C] phenylalanine. This experiment revealed that [2-^13^C] phenylacetyl-CoA accumulated within the *ΔpaaC* mutant with no significant accumulation of [2-^13^C] phenylpyruvate, supporting the hypothesis that Ior1 converts phenylpyruvate to phenylacetyl-CoA. PaaABCDE subsequently catalyzes the formation of an epoxide on the phenyl ring of phenylacetyl-CoA, which is then converted to a 7-membered cyclic ether by PaaG [59]. This ether ring is then opened by the activity of PaaZ, a bifunctional enzyme that contains enoyl-CoA hydratase and aldehyde dehydrogenase domains. The hydratase domain catalyzes an addition of water to the ether ring forming a hemiacetal that undergoes ring-opening to form 3-oxo-5,6-dehydro-suberoyl-CoA, the precursor to the tropone ring.

## 3. Gammaproteobacteria

**Figure 3:**
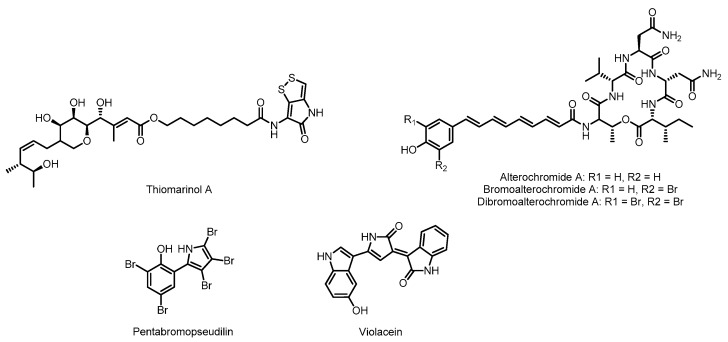
Natural products from marine gammaproteobacteria with characterized biosynthetic pathways.

Gammaproteobacteria is one of the most diverse classes of bacteria, second only in number of genera to the Firmicutes [25]. This class includes some of the most well known human pathogens, the Enterobacteriales, whose members include *Escherichia coli, Salmonella enterica,* and *Yersinia pestis.* The marine members of the gammaproteobacteria class have been an especially productive source of natural products, particularly the genus *Pseudoalteromonas* [60]. The marine gammaproteobacteria are known to produce a number of metabolites with fascinating chemistry such as the oxazolinone, indolomycin [61], whose synthesis involves the PLP-mediated oxidation of unactivated carbon-carbon bonds [62], or the beta-amino acid and beta-keto acid containing peptide product andrimid [63,64,65]. These molecules are also produced by *Streptomyces*, where their biosyntheses have been interrogated; as a result, they will not be discussed further in this review. With such a diversity of species and environmental conditions, it is not surprising that the marine gammaproteobacteria are a rich source of natural product diversity. Thiomarinol, the alterochromides, pentabromopseudilin, and violacein (see Figure 3) are examples of gammaproteobacterial natural products that have been linked to their gene cluster.

### 3.1. Thiomarinol

Thiomarinol and its derivatives are produced by the marine gammaproteobacterium *Pseudoalteromonas* sp. SANK 73390 and show strong antibiotic activity against a number of drug resistant pathogens, including multidrug resistant *Staphylococcus aureus* 507 (MRSA) [66]. Thiomarinol is a hybrid molecule composed of an NRPS-derived dithiopyrrolone (DTP) group and a polyketide based marinolic acid group. The genetic elements responsible for the production of thiomarinol have been identified through the characterization of a naturally occurring plasmid containing the *tml* gene cluster [67].

**Scheme 4:**
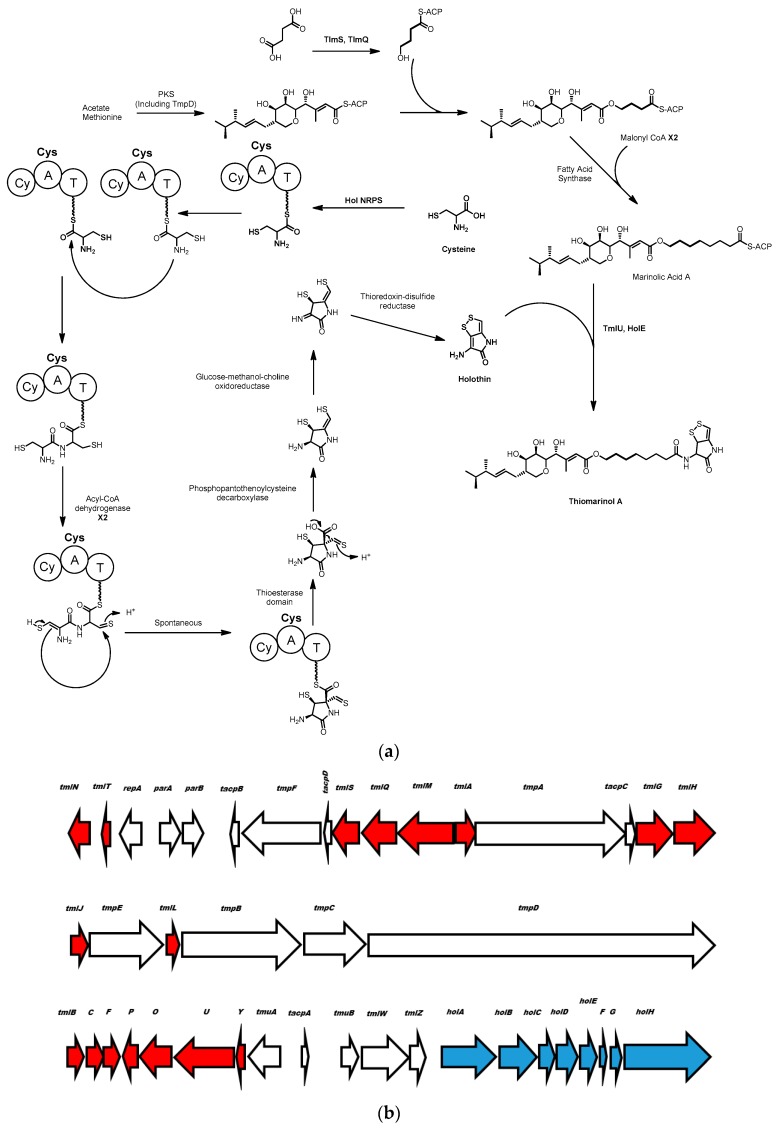
(**a**) Proposed biosynthesis of thiomarinol from holothin and marinolic acid residues catalyzed by TmlU and HolE. Cy = cyclization, A = adenylation, T = thiolation; (**b**) the structure of the thiomarinol biosynthetic gene cluster. Genes for the synthesis of marinolic acid are shown in red, genes for the synthesis of holothin are shown in blue.

It has been known since the 1950s that the DTP motif found in thiomarinol is also used in natural products such as thiolutin, aureothricin, holothin and holomycin produced by terrestrial *Streptomyces* species [68,69]. Li and Walsh proposed a biosynthetic route to DTP (see Scheme 4) based on disruption of the holomycin gene cluster in *Streptomyces clavuligerus*, in addition to purification and in vitro characterization of the enzymes encoded by this cluster [70]. A gene knockout identified a single NRPS module containing cyclization, adenylation and peptide carrier protein domains that is essential for production of holomycin. The authors propose the biosynthesis could proceed by loading of cysteine onto the carrier protein followed by condensation between two NRPS-bound cysteines. Subsequently two oxidation steps by an acyl-CoA dehydrogenase would convert the two thiols to an ene-thiol and a thial, setting up a non-enzymatic cyclization to form the pyrrolidone by nucleophilic attack of the enethiol on the thial (see Scheme 4). Following thioesterase mediated release of the molecule from the NRPS machinery, decarboxylation by a predicted phosphopanthenoylcysteine decarboxylase is proposed, facilitated by the electron sink properties of a beta thialdehyde. Finally, disulfide formation generates the dithiopyrrolone moiety, holothin.

The synthesis of the marinolic acid portion of thiomarinol is shown in Scheme 4 and is similar to how the mupirocin class of molecules is produced in the non-marine gammaproteobacterium, *Pseudomonas fluorescens*, which has been investigated in some detail [71]. Based on the similarity in the structure of the gene clusters responsible for mupirocin synthesis in *Pseudomonas* and *Pseudoalteromonas,* it is proposed that the biosynthesis proceeds through the same mechanism in both genera [67] Mupirocin is the name given to a collection of hybrid polyketide-fatty acid molecules, principally pseudomonic acids A-D, with differing fatty acid chain lengths and degree of polyketide tailoring (reviewed thoroughly in [72]). Mupirocin has several unique features amongst polyketides, principally that its synthesis relies on *trans*-acting acyltransferase and enoyl reductase domains encoded in the genome distal from the rest of the mupirocin gene cluster [73]. Also of interest is that methyl groups are added to malonate residues after their incorporation into mupirocin by an *S*-adenosyl methionine methyltransferase and an HMG-CoA synthase, rather than acyl-transferase domain selection of methyl-malonyl-CoA [74]. There is also no apparent loading module for *Pseudomonas* mupirocin production, unlike many PKS systems that have specific enzymes responsible for the loading of a starter unit onto an acyl carrier protein to initiate metabolite production [13]. Experiments where *Pseudoalteromonas* sp. SANK 733390 was fed with [^13^C]-labeled carbon sources show that the substrates for the polyketide machinery of marinolic acid synthesis, like mupirocin synthesis, are derived from acetate, 4-hydroxy butyrate or succinate, and methionine [75].

There are clear analogues of the genes coding for holothin and mupirocin biosynthetic enzymes in *Pseudoalteromonas* sp. SANK 733390 borne on the naturally occurring plasmid pTML1 [67]. Mutants with inactive *holA,* (NRPS used in holothin biosynthesis), or ketosynthase *tmpD* (used in marinolic acid production), were incapable of thiomarinol synthesis. However, these mutations can be complimented by addition of exogenous mupirocin or holothin, confirming their roles as building blocks for thiomarinol biosynthesis [75,76]. This same study [76] indicated that the *holA* deficient mutant could incorporate amines such as anhydro-ornithine into the thiomarinol backbone to create synthetic variants of thiomarinol.

Although much of the thiomarinol cluster is composed of genes that have analogues in the mupirocin/marinolic acid cluster from *Pseudomonas fluorescens,* or the DTP holothin cluster from *Streptomyces clavuligerus*, several genes are unique to *Pseudoalteromonas* sp. SANK 73390. Using in vitro experiments, Li and co-workers showed that two of these enzymes (TmlU and HolE) are involved in the key step, unique to thiomarinol biosynthesis, involving coupling of the dithiopyrrolone and marinolic acid groups. In vitro characterization of TmlU and HolE [77] reveals that TmlU ligates free marinolic acid to coenzyme A and then HolE is responsible for coupling the holothin and marinolic acid-CoA substrates together to form thiomarinol. These in vitro analyses also reveal a high degree of substrate promiscuity for both enzymes. TmlU can ligate a wide range of acyl carboxylic acids to coenzyme A, from pseudomonic acid, a close analogue of marinolic acid, to octanoic acid and acetic acid. Additionally, HolE was also capable of ligating holothin to a wide range of acyl-CoA molecules such as butanoyl-CoA and palmitoyl-CoA. HolE was even capable of ligating non-holothin amines such as homoserine lactone and 3-aminocoumarin to pseudomonic acid (the analogue to marinolic acid). The reactions with alternative substrates all proceed with relatively high efficiency, with between 10% and 100% of the activity seen with the natural substrates. This substrate promiscuity creates an interesting opportunity for the combinatorial biosynthesis of novel antibiotic compounds. Thiomarinol is a naturally occurring hybrid antibiotic composed of two pharmacophores, the marinolic acid and DTP/holothin moieties, with different modes of activity.

The pseudomonic/marinolic acid is an inhibitor of isoleucyl-tRNA synthase [78] whereas DTP antibiotics are inhibitors of RNA synthesis in Gram-negative bacteria [79], and the combination of these two activities in thiomarinol generates a more potent antibiotic than either alone [66]. Due to the promiscuity of TmlU and HolE, there is potential to generate any number of hybrid antibiotics exploiting this amide ligation mechanism.

### 3.2. Alterochromides

The alterochromides are produced by multiple *Pseudoalteromonas* species, the molecules contain a *C*-terminal cyclic pentapeptide composed of TVNNL/I [80] and an *N*-terminal unsaturated fatty acid moiety terminated by a phenolic group. The latter is brominated in several members [80]. Brominated alterochromides show cytotoxicity against sea urchin embryos and moderate antibiotic activity against Gram-positive bacteria [80,81].

The ~34 kb *alt* biosynthetic gene cluster encoding the synthesis of this lipopeptide from *Pseudoalteromonas piscicida* JCM 20779 was predicted by genomic analysis using antiSMASH [30,82]. The cluster was cloned in its entirety using a targeted capture approach and heterologously expressed in *E. coli* [49]. This has led to direct observations of the role of several genes in the *alt* cluster for the synthesis of alterochromides. MS based molecular networking has facilitated identification of a series of alterochromide molecules produced by *P. piscicida* JCM 20779, with minor variations in amino acid selectivity, fatty acid chain length, and degree of bromination [82].

**Scheme 5:**
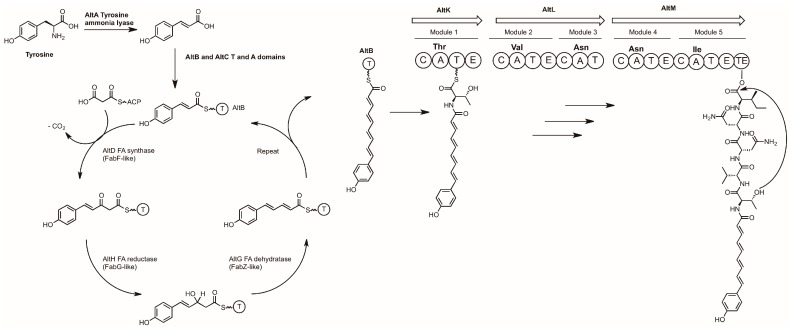
Proposed biosynthesis of the alterochromides. A = Adenylation domain, C = Condensation domain, T = Thiolation domain, E = Epimerization domain, TE = Thioesterase domain.

Targeted knockouts of the cloned *alt* cluster has allowed for a biosynthetic pathway to be proposed (Scheme 5).

AltA catalyzes the first committed step of this pathway, converting tyrosine to coumaric acid, the starter unit for the fatty acid synthase. All molecule production was abolished in the *ΔaltA* knockout strain, while complementing the mutant with coumaric acid fully restored production of the alterochromides. Coumaric acid is tethered to the acyl carrier protein AltB to enable polyene synthesis by the sequential activities of AltD (condensation with malonyl-CoA), AltH (reduction), and AltG (dehydration). The fatty acid is transferred to the NRPS, where the peptide portion of the alterochromides is synthesized in a collinear manner and then cyclized by the thioesterase domain of AltM. To date the timing and substrate for the bromination step is unknown, but deletion of the *altN* gene abolishes the production of brominated alterochromides while still allowing for the synthesis of the non-brominated analogues.

### 3.3. Pentabromopseudilin

Pentabromopseudillin (see Figure 3) is composed of a brominated phenol coupled to a brominated pyrrole and was first isolated from a marine pseudoalteromonad in the 1960s [83,84]. Pentabromopseudilin has long been known to have moderate antibiotic activity [83], and a class of structurally similar molecules known as the polybrominated diphenyl ethers (PBDEs) have been proposed as potential broad-spectrum antibiotics due to their ability to inhibit DNA and protein synthesis in both Gram-positive and Gram-negative pathogens, albeit with some cytotoxic effects in mammalian cells [85,86]. Pentabromopseudilin and the PBDEs have closely related biosynthetic origins, the detailed understanding of which has been elusive until recently.

Feeding assays in the 1990s and early 2000s with ^13^C-labeled amino acids have shown that the pyrrole ring of pentabromopseudilin comes from proline [87] while the phenyl ring is derived from *para*-hydroxybenzoic acid via the chorismate pathway [88]. The genetic basis for the synthesis of pentabromopseudilin (and other brominated phenolic compounds) was determined through genomic prediction of gene function, heterologous expression, in vivo gene inactivation, and in vitro characterization of the biosynthetic enzymes. The genomes of *Pseudoalteromonas* strains that produce bromopyrroles, *P. luteoviolacea* 2ta16 and *P. phenolica* O-BC30, were sequenced and queried with genes from the pyoluteorin biosynthetic gene cluster from the soil associated gammaproteobacterium *Pseudomonas fluorescens* Pf-5 [89].

**Scheme 6:**
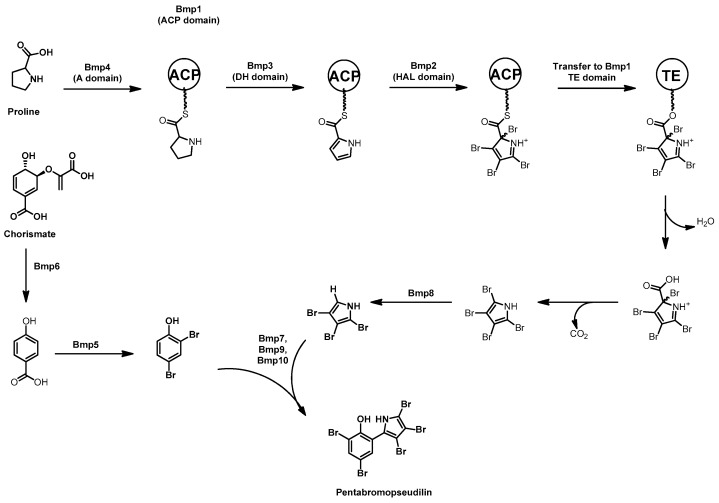
Pentabromopseudilin biosynthesis showing the synthesis of the bromopyrrole from proline, and the bromophenol from chorismate. A = adenyltransferase, ACP = Acyl carrier protein, DH = flavin-dependent dehydrogenase, Hal = flavin-dependent halogenase, TE = Thioesterase domain.

Putative homologues to the proline dehydrogenase (*pltE)* and pyrrole halogenase (*pltA*) genes encoding production of the chlorinated hetero-bipyrrole pyoluteorin were discovered in the genomes of the two *Pseudoalteromonas* strains alongside additional putative biosynthetic genes. Heterologous expression of the putative pentabromopseudilin biosynthetic gene cluster, *bmp1–8*, in *E. coli* confirmed the correct gene cluster had been identified [90,91]. Serial deletions of plasmid borne *bmp1–7* were then undertaken to identify the biosynthetic role of each enzyme in the pathway. These in vivo experiments coupled with in vitro enzyme assays of the products of the *bmp* cluster have provided a detailed picture of PBDE and pentabromopseudilin synthesis, which is summarized in Scheme 6. Each of the biosynthetic genes have been expressed in *E. coli*, the enzymes purified, and their activities characterized in vitro. Indeed, the entire biosynthetic pathway has been reconstituted in vitro by sequential addition of each purified enzyme providing insight into how brominated phenols, bromopyrroles, homo and heterodimers of these two, as well as polybrominated dioxins are produced by the *bmp* pathway [92,93]. The bromopyrrole moiety is synthesized by Bmp1–4. Firstly, Bmp4, a proline adenyltransferase, loads proline onto the ACP domain of Bmp1, then Bmp3, a flavin-dependent dehydrogenase, oxidizes the proline to form an ACP-bound pyrrole. This is followed by tetra-bromination by Bmp2, a flavin-dependent halogenase [94]. The introduction of four halogens onto a single pyrrole is atypical behavior for flavin-dependent halogenases, where 1–2 halogens is the norm.

**Figure 4:**
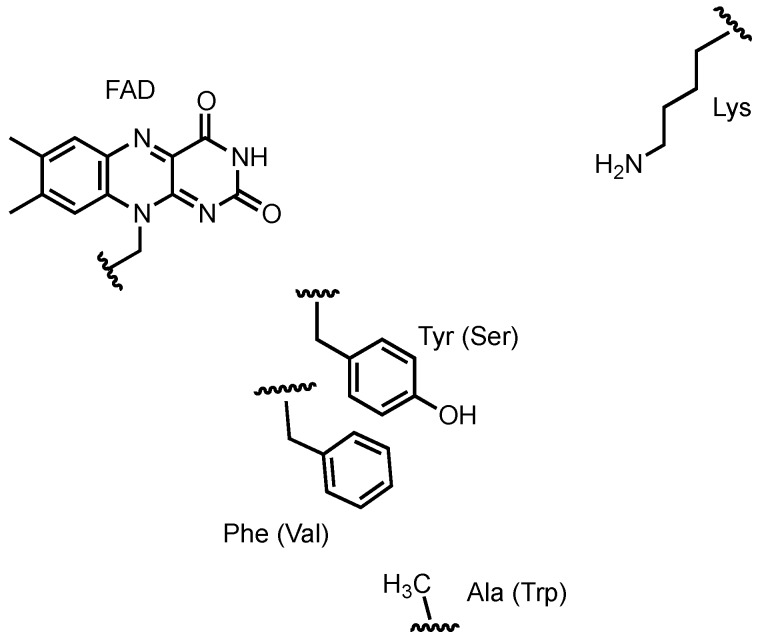
Residues in the putative pyrrole-binding active site of halogenase Bmp2. Residues in structurally equivalent positions in Mpy16 and in mutant Bmp2 are named in parentheses. Active site figure based upon crystal structures reported in [94].

To probe this behavior, the X-ray crystal structures of Bmp2 and Mpy16, a flavin-dependent dichlorinase from marinopyrrole A biosynthesis [95], were compared. Although no co-structures with bound substrate were obtained, the location of pyrrole binding was modeled and the relative position in the active site of the catalytic lysine and FAD isoalloxazine remain constant. Three non-conserved residues in the Bmp2 active site were identified and mutated to the corresponding residues from Mpy16 (Y302S/F306V/A345W) resulting in a mutant with mono-halogenating behavior (see Figure 4). Intriguingly, the Bmp2 mutant remained selective for bromide, a phenomenon that is not understood at this point. Following bromination of the pyrrole, hydrolytic offloading generates an acid that spontaneously decarboxylates to restore aromaticity, resulting in a tetra-brominated pyrrole. The 2′position bromine is reductively removed through the activity of Bmp8, resulting in a tribromopyrrole that is then incorporated into pentabromopseudilin [96]. It is proposed that reductive dehalogenation occurs via the oxidation of cysteine residues of Bmp8 to form a disulfide and HBr.

The second building block for pentabromopseudilin is the dibromophenol moiety, its synthesis begins with a chorismate lyase, Bmp6, that converts chorismate to *para*-hydroxybenzoic acid, which is then *ortho* and *para* brominated by the flavin-dependent brominase Bmp5, resulting in the loss of CO_2_ and the formation of dibromophenol (see Scheme 6) [93]. The two brominated aromatic compounds are then coupled together by the promiscuous P450 enzyme Bmp7 with the assistance of ferredoxin (Bmp9) and ferredoxin reductase (Bmp10). Bmp7 mediates radical coupling of both bromopyrrole and bromophenol, resulting in homodimers, the heterodimer pentabromopseudillin, polybrominated ethers, and polybrominated dioxins [93].

The recent investigations of the pentabromopseudilin pathway are an excellent example of why marine proteobacteria are such fruitful sources of biosynthetic information. Detailed characterization of the activities and substrate selectivity of each Bmp enzyme and the near complete in vitro reconstitution of the biosynthesis was aided greatly by facile heterologous expression in *E. coli.* This approach as compared to more traditional in vivo methods allows a great deal of control over each experiment and facilitates a deeper interrogation of the chemical mechanisms.

### 3.4. Violacein

**Scheme 7:**
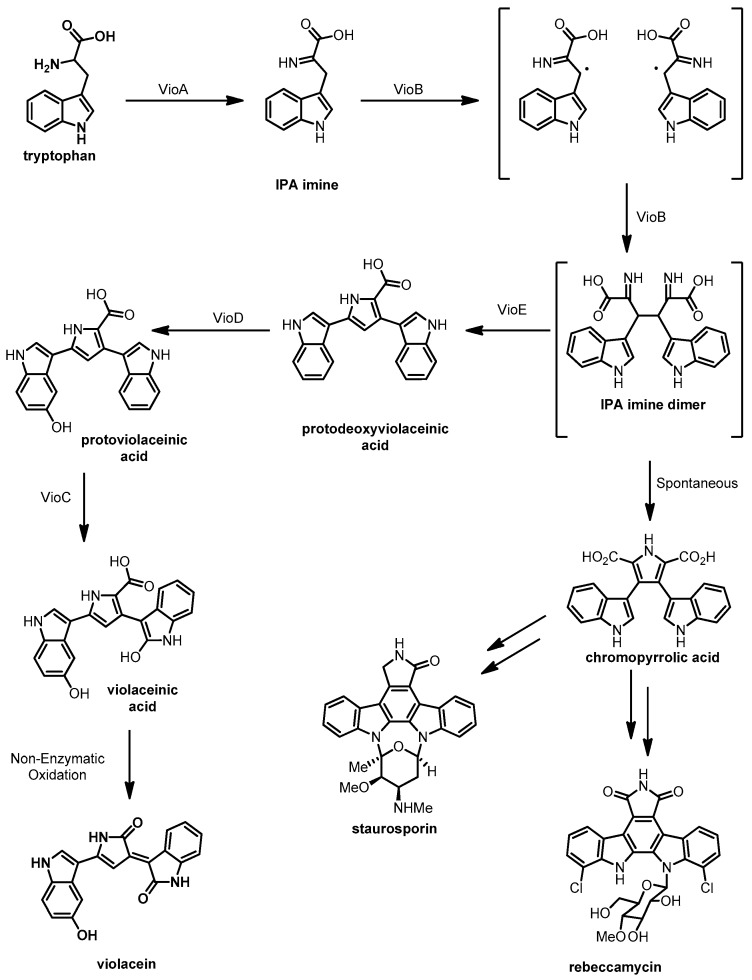
Biosynthetic route for violacein proposed by Shinoda et al. [113].

Violacein is a purple indolocarbazole pigment that was discovered in the 1880s and has been isolated from a range of Gram-negative bacterial species such as the terrestrial betaproteobacterium *Chromobacterium violaceum* [97,98] the amphibian commensal betaproteobacterium *Janthinobacterium lividum* [99] and the soil associated betaproteobacterium *Dunganella* sp. [100,101]. It is also synthesized by numerous marine gammaproteobacteria including *Pseudoalteromonas luteoviolacea* [102], and *Pseudoalteromonas* sp. 520P1 [103]. Violacein has antibacterial and antiviral activities and cytotoxic effects against MOLT-4 leukemia and NCI-H460 lung tumor cells [104]. The biosynthesis of violacein is shown in Scheme 7 and involves the oxidative coupling of two L-tryptophan molecules catalyzed by enzymes encoded in the *vio* gene cluster [105].

The biosynthesis of violacein by terrestrial *Chromobacterium* species has been investigated for several decades, as discussed in a 2011 review and the references within [106]. By way of an overview we will highlight a few of these studies. An initial heterologous expression of the *vio* gene cluster in *E. coli* delineated the borders of the genetic locus required for violacein biosynthesis. Subsequently using in vitro assays, Balibar and Walsh reconstituted VioA–E from *Chromobacterium violaceum* and found the 5 enzymes were sufficient for synthesis of violacein from L-Trp [107]. The flavin-dependent amine oxidase VioA and the heme-dependent oxidase VioB work together to oxidize L-tryptophan and dimerize the resultant imine of indolepyruvic acid (IPA imine) via a radical coupling mechanism [108]. VioE catalyzes a rearrangement of the IPA imine dimer to form protodeoxyviolaceinic acid; despite the publication of the crystal structure of VioE in 2008, the exact mechanism of this step remains unclear [109,110]. The IPA imine dimer intermediate is an important branch point in the biosynthesis of many indolocarbazole molecules; in pathways that lack a VioE homologue the IPA imine dimer spontaneously forms chromopyrrolic acid that can be further modified to generate natural products such as rebeccamycin and staurosporine [111,112].

The final two enzymes encoded by the *vio* cluster, VioC and VioD, are flavin-dependent mono-oxygenases that require NADH/NADPH as a reductive co-factor. VioD first hydoxylates one indole ring at the 5 position to form protoviolaceinic acid and then VioC oxidizes the other indole ring at the 2 position to form hydroxyindole. The oxidation sequence of VioC and VioD was demonstrated by Shinoda et al. [113] by isolating protoviolaceinic acid from a reaction of tryptophan, NADPH, and VioABDE, and showing that this product was converted by VioC in the presence of NADPH. The final step of the biosynthesis is a spontaneous decarboxylation and oxidation of the hydroxyindole to form violacein.

There has been limited investigation of the biosynthesis of violacein in the marine *Pseudoalteromonas* species. Zhang and Enomoto [103] identified a potential gene cluster in *Pseudoalteromonas* sp. 520P1, based on homology to the *C. violaceum* locus. They probed a *Pseudoalteromonas* 520P1 fosmid library for homologues of *vioC* from *C. violaceum* and a 7383 bp clone was identified that contained *vioA–E* aligned in a single operon with 52.8% homology to *vioA–E* from C. violaceum. Initial attempts to express the *P.* sp. 520P1 violacein cluster from the unaltered fosmid in *E. coli* were unsuccessful.

The authors had previously noted the involvement of *N*-(3-oxooctanoyl)-homoserine lactone in regulation of violacein production in *P.* sp. 520P1 [114] and thus hypothesized that expression of the *vio* cluster in *E. coli* failed due to the absence of the requisite genes for synthesis and recognition of the homoserine lactone. The authors then cloned *vioA–E* into a recombinant pET vector in front of a T7 promoter, effectively replacing the native upstream promoter region of the violacein biosynthetic cluster, this modification resulted in production of violacein by *E. coli*. Although the biosynthetic enzymes required to produce violacein in *Chromobacterium* and *Pseudoalteromonas* strains are very similar, there are differences in how the biosynthesis is regulated. Whilst *Pseudoalteromonas* sp. 520P1 needs an AHL for production of violacein [114], *Pseudoalteromonas ulvae* TC14 is capable of producing violacein without AHLs and does not appear to make them intrinsically, however, addition of different exogenous AHLs to *P. ulvae* TC14 can increase or decrease production levels of violacein [115].

The variable regulation of violacein production in *Chromobacterium* and *Pseudoalteromonas* demonstrates a major challenge facing natural product discovery through heterologous expression. Regulatory elements of a gene cluster must be considered and genetic manipulations may be needed to achieve heterologous expression. Fortunately, the genetic tractability of *E. coli* allows for relatively facile modification of regulatory elements, however, our understanding of pathway regulation in proteobacteria is limited and there is much still to learn.

**Figure 5:**
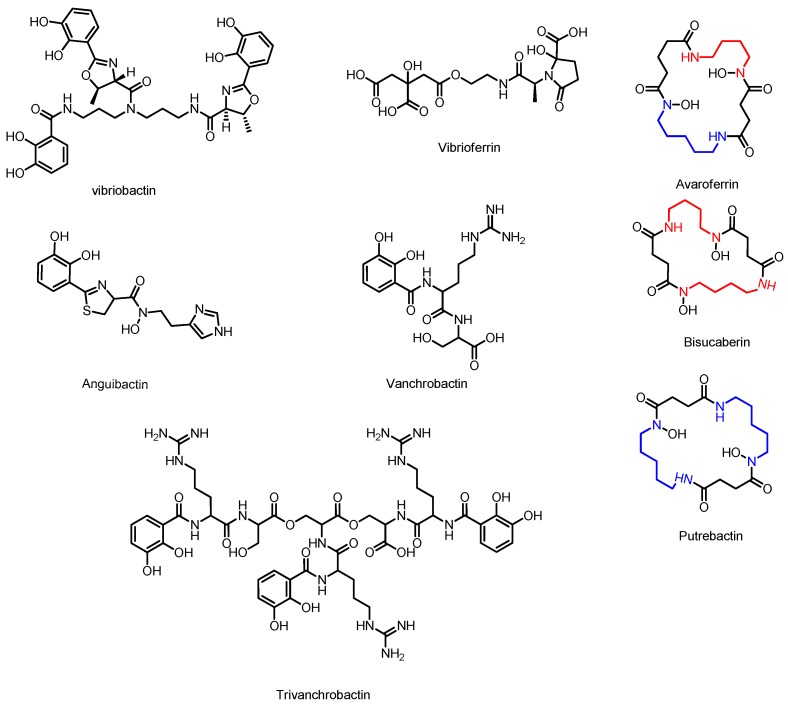
Marine gammaproteobacterial siderophores with characterized biosynthetic gene clusters.

Siderophores represent a considerable number of the natural products produced by marine gammaproteobacteria that have been linked experimentally to their respective gene clusters (see Figure 5). Siderophores are iron-chelating molecules that facilitate the import of environmental iron (III) into the cell [116,117]. Marine microorganisms are prolific siderophore producers due to the very low concentrations of bioavailable iron in seawater [118].

### 3.5. Avaroferrin, Putrebactin, and Bisucaberin

Avaroferrin, putrebactin and bisucaberin are gammaproteobacterial hydroxamate siderophores that inhibit the swarming behavior of *Vibrio* species [119]. These siderophores are structurally derived from two *N*-hydroxyl-*N*-succinyl diamines (NSD), *N*-succinyl *N*-hydroxy cadaverine (SHC) and *N*-succinyl *N*-hydroxy putrescine (SHP) [119]. Avaroferrin from *Shewanella algae* is a heterodimer of the two NSDs, Bisucaberin from *Pseudoalteromonas haloplanktis* and *Vibrio salmonicida* is a homodimer of SHC and putrebactin from *Shewanella putrefaciens* is a homodimer of SHP.

**Scheme 8:**
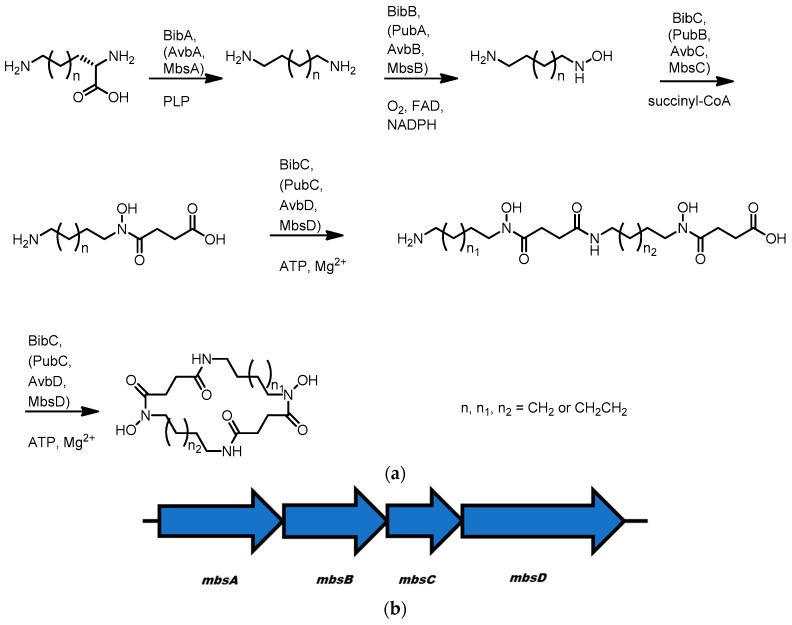
(**a**) General outline of the biosynthesis of NSD-derived siderophores avaroferrin, bisucaberin, and putrebactin; (**b**) the *mbs* gene cluster from metagenomic DNA that encodes the synthesis all three molecules.

It appears that all three molecules are made in an analogous fashion to the well-characterized siderophore desferrioxamine produced by Streptomyces [120,121,122,123]. The biosyntheses involve four chemical steps (Scheme 8); firstly, pyridoxal phosphate-dependent decarboxylation of ornithine or lysine to form putrescine and cadaverine, respectively, subsequent mono-hydroxylation of these diamine molecules by an FAD-dependent monoamine oxidase, succinylation of the resultant hydroxylamine with succinyl CoA and then ATP and magnesium-dependent dimerization/cyclization by a siderophore synthetase to form the mature siderophore. Whilst an exhaustive investigation of each molecule’s biosynthesis has not been completed, elements of each pathway have been studied.

Early work by Challis and co-workers identified the putative gene clusters *bibA–C* and *pubA–C* for bisucaberin and putrebactin in *Vibrio salmonicida and Shewanella putrefaciens* respectively. These pathways were identified by searching for homologues of DesD, the characterized ATP-dependent trimerization/cyclization enzyme in the biosynthetic pathway to desferrioxamine E [123]. To interrogate the *bib* and *pub* gene clusters, the DesD homologues BibC and PubC were expressed and purified. These enzymes were shown to convert SHC and SHP to bisucaberin and putrebactin in an ATP-dependent manner [124,125]. The remaining genes in the gene clusters resembled those in the *des* pathway, and therefore analogous functions were assumed.

Similar molecules and gene clusters have been identified in a number of other bacteria. Avaroferrin was identified along with a homologous gene cluster *avbA–D* in *Shewanella algae.* Closer inspection of *S. algae* extracts also showed production of bisucaberin and putrebactin in addition to avaroferrin, suggesting the same pathway can produce all three molecules and the biosynthetic enzymes must accept several substrates [119]. Soe et al. obtained similar results when they inhibited the function of the ornithine decarboxylase in *S. putrefaciens* and fed alternative diamine building blocks resulting in formation of avaroferrin and bisucaberin in addition to putrebactin [126]. Finally, a homologous gene cluster of unknown bacterial origin has been identified in a deep-sea sediment metagenomic library [127]. The gene cluster was discovered using the chromazurol S (CAS) assay to screen library members for iron chelation behavior. Four genes were identified; *mbsA–D* and heterologous expression of the pathway in *E. coli* resulted in production of all three siderophores. As in the case of the Avb pathway, the Mbs enzymes appear to accept multiple substrates as both SHC and SHP could be detected in cultures of *E. coli* harboring plasmids bearing *mbsA-C* [128]. This same study also implicates MbsD as responsible for the final macrocyclization step of avaroferrin/bisucaberin/putrebactin biosynthesis as co-expression of MbsA–C and the *C*-terminal portion of DfoC, an MbsD homologue from the desferrioxamine producer *Erwinia amylovora*, results in the production of desferrioxamine E instead of the *Shewanella* siderophores.

Of the gene clusters identified thus far, *mbs* and *avb* are composed of 4 open reading frames A–D, *bib* is composed of three ORFs where *bibC* appears to be a fusion ORF homologous to *mbsC* and *mbsD*. The *pub* locus is also composed of three ORFs however in this case the ornithine decarboxylase is absent and is likely located external to the main cluster. Despite their architectural differences, most of these pathways appear capable of producing multiple molecules given the right conditions suggesting the biosynthetic enzymes have an inherent promiscuity allowing formation of structural diversity.

### 3.6. Vibriobactin

Vibriobactin is a catecholate siderophore produced by *Vibrio cholerae*, the pathogenic bacterium responsible for causing Cholera [129]. This NRPS derived peptide is constructed from a molecule of norspermidine (NSPD), three dihydroxy benzoic acids (DHB), and two threonine residues. Extensive investigations of the biosynthesis of this molecule have been completed using a combination of in vivo and in vitro experiments.

**Scheme 9:**
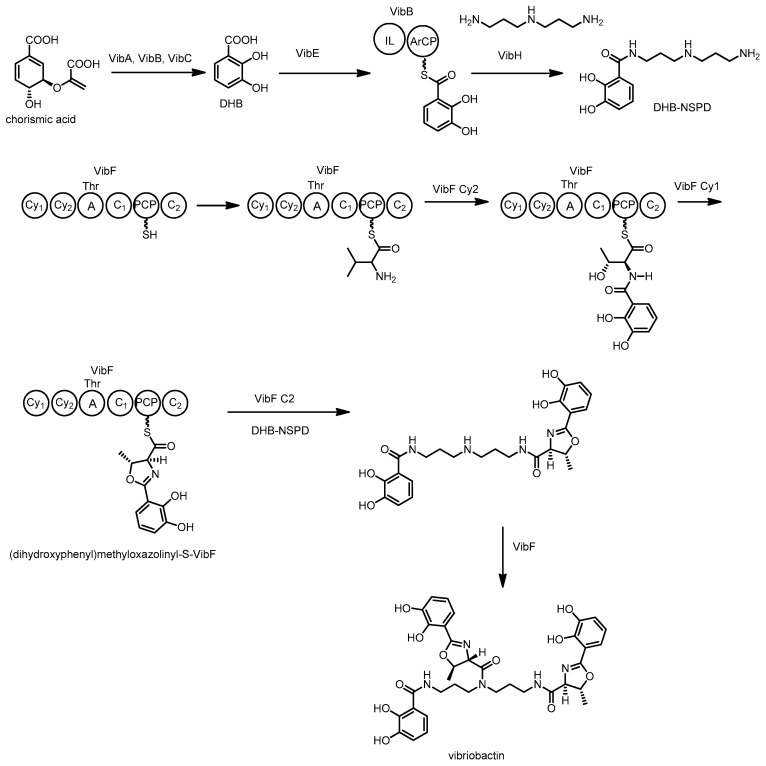
Biosynthesis of vibriobactin by *Vibrio cholerae*. C = condensation, Cy = cyclization, A = adenylation, ArCP = aryl carrier protein, PCP = peptidyl carrier protein, IL = isochorismate lyase.

Early efforts using gene inactivation and in vivo analysis identified that the genes required for biosynthesis and transport of vibriobactin reside in two separate loci on the chromosome of *Vibrio cholera* [130,131,132,133,134]. Via genetic experiments, *vibA*, *vibB* and *vibC* were found responsible for DHB production, while *vibB*, *vibE*, *vibF* and *vibH* are needed for the synthesis of vibriobactin from DHB and norspermidine [130,132,133]. Subsequent studies by Walsh and co-workers with the encoded enzymes clarified many of the biosynthetic steps. In 2000 they described the in vitro biosynthesis of vibriobactin by VibBEFH using DHB and NSPD in the presence of ATP [135,136]. Firstly, DHB is adenylated by VibE and loaded onto the aryl carrier protein (ArCP) domain of VibB (see Scheme 9). VibH, a standalone homologue of a condensation domain, catalyzes the *N*-acylation of free NSPD with DHB-VibB [135,137], generating free NSPD-DHB. VibH is somewhat unusual when compared to condensation domains found in other NRPS systems, as it accepts free substrates rather than the more typical carrier protein bound substrates.

The second part of the biosynthesis uses VibF, an NRPS consisting of six domains, annotated as two cyclization, two condensation, an adenylation, and a peptidyl carrier protein domain. The adenylation domain activates threonine and loads it onto the VibF carrier protein, followed by amide bond formation with VibB bound DHB to form Thr-DHB. VibF then catalyzes cyclization of the threonine hydroxyl group with the amide carbonyl, followed by dehydration to form an enzyme-bound dihydroxyphenyl-methyloxazoline. Finally vibriobactin is produced by acylation of DHB-NSPD with two equivalents of dihydroxyphenyl-methyloxazoline [136].

Site directed mutagenesis and whole domain deletion experiments followed by in vitro characterization of enzyme-generated products determined that the two cyclization domains of VibF serve distinct roles in the production of the methyloxazoline moiety of vibriobactin. Cy2 acts to condense together the PCP bound threonine and ArCP bound DHB and then Cy1 catalyzes the heterocyclization and dehydration reactions [137]. This process is highly unusual, as many cyclization domains from other pathways such as those to produce yersiniabactin [138], epothilone [139] and mycobactin [140] perform all three steps with one enzyme.

The functions of the two condensation domains of VibF were also examined in vitro. C2 was found to catalyze reactions between NSPD-DHB and two equivalents of (dihydroxyphenyl) methyloxazolinyl-S-VibF to generate vibriobactin. C1 was not responsible for any catalytic steps [141] and subsequent investigations found the VibF exists as a dimer and C1 functions to improve the binding between monomers [142,143]. Heterodimers of different VibF mutants could restore function lost in the individual mutants suggesting several domains act on the acyl intermediates *in trans*.

### 3.7. Anguibactin and Vanchrobactin

Anguibactin and Vanchrobactin are two catecholate peptide siderophores produced by marine *Vibrio* species. These molecules are key virulence factors for the fish pathogen *V. anguillarum* [144,145]. A promising aquaculture antibiotic has been developed based on synthetic conjugates of the antibiotic norfloxacin and vanchrobactin derivatives, based on the hypothesis that siderophore uptake can overcome antibiotic resistance through efflux [146]. Anguibactin is also uncommon amongst microbial siderophores for its ability to complex with gallium(III) in addition to iron [147]. The gene cluster responsible for synthesis of anguibactin has been discovered on the virulence-associated plasmid pJM1 [148], however, this cluster is also found in the chromosomes of some *Vibrio* strains in association with the gene cluster responsible for vanchrobactin biosynthesis [149]. Anguibactin is composed of a histamine residue, a thiazole ring derived from cysteine, and dihydroxy benzoic acid, whereas vanchrobactin is a linear peptide composed of serine, arginine, and dihydroxy benzoic acid. Divanchrobactin and trivanchrobactin are linear dimers and trimers of vanchrobactin synthesized by the same producer [150]. Enzymes responsible for the synthesis of the DHB residue from chorismate are encoded by both gene clusters in the form of *angB/G* (an isochorismate lyase/aryl carrier protein), *angC* (isochorismate synthase), and *angE* (2,3-dihydroxybenzoate-AMP ligase) and their functional homologues *vabB*, *vabC*, and *vabE* respectively.

**Scheme 10:**
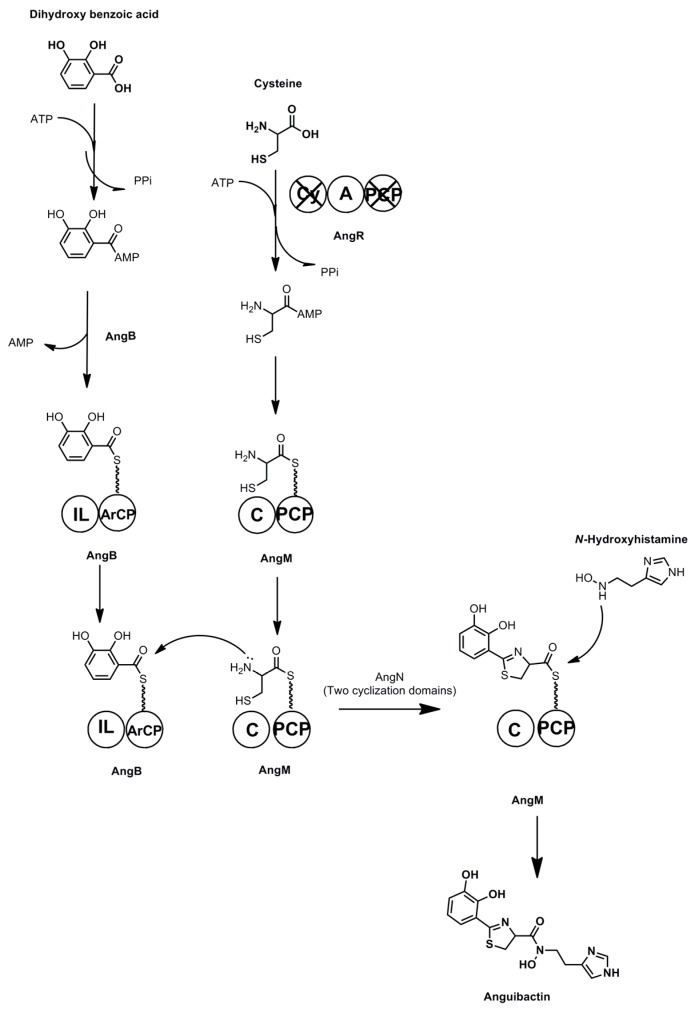
Anguibactin biosynthesis. C = condensation, A = adenylation, ArCP = aryl carrier protein, PCP = peptidyl carrier protein, IL = isochorismate lyase, Cy = cyclization. Non-functional domains are marked with an X.

NRPS driven anguibactin biosynthesis begins with loading of DHB onto the phosphopantetheinylated ArCP module of AngB (see Scheme 10). The adenylation domain of AngR activates a cysteine molecule and then loads it onto the PCP domain of AngM. AngB-bound DHB is then coupled to AngM-bound cysteine, and the thiazole moiety is formed by the two cyclization domains of AngN [149]. An *N*-hydroxy-histamine residue is produced through the activities of the AngH and AngU proteins that decarboxylate and then hydroxylate histidine. This free *N*-hydroxy histamine residue is coupled to the AngM bound peptide by the action of a condensation domain, and this liberates the peptide from the carrier protein, resulting in formation of anguibactin.

Several of the biosynthetic enzymes in this pathway are bi-functional, and demonstrate unusual features. Expression of the *angB/G* locus in DHB deficient strains of *E. coli* followed by protein microsequencing reveals that one genetic locus is responsible for the production of two proteins, AngB and AngG, with related functions [151]. The amino terminal domain of AngB has isochorismate lyase activity, whereas the carboxy terminal domain of AngB is an ArCP facilitating non-ribosomal peptide synthesis. AngG is translated in the same frame as AngB, but is composed of only the *C*-terminal ArCP domain. The specific role of AngG in anguibactin biosynthesis is unclear, however, it may serve as an alternative ArCP to that found in AngB.

Another multifunctional enzyme involved in anguibactin biosynthesis is AngR. Transposon mutagenesis of *Vibrio anguillarum* followed by RNAse protection assays shows that the *N*-terminal domain of AngR, a helix-turn-helix leucine zipper domain, is essential for the transcription of the iron transporter gene *fatB* [152]. The *C*-terminal domain of AngR contains NRPS type modules, with apparently non-functional cyclization and peptidyl carrier protein domains and a functional adenylation domain responsible for adenylating a serine molecule prior to its incorporation into the anguibactin product.

Vanchrobactin is synthesized using an analogous NRPS-type biosynthetic pathway encoded by the *vab* gene cluster. Firstly, DHB is loaded onto the ArCP module of VabB [153], secondly VabF, a multidomain NRPS composed of two condensation, two adenylation, two PCP, and a thioesterase domain acts to incorporate arginine and serine into the molecule by typical NRPS extensions and finally hydrolytic offloading yields vanchrobactin. Tandem mass spectral analysis of cultures of *Vibrio* sp. DS40M4 have also identified trivanchrobactin, a linear trimer of the vanchrobactin peptide formed through serine ester linkages [150]. It is hypothesized that iterative functioning of the assemblyline would result in formation of the trimer, the exact role of the VabF thioesterase in this polymerization is still to be elucidated.

### 3.8. Vibrioferrin

Vibrioferrin (see Figure 5) is a carboxylate class siderophore produced by the marine pathogen *Vibrio parahaemolyticus* [154]. Vibrioferrin is notable for having one of the weakest binding affinities for iron and the greatest susceptibility to photolysis of all marine siderophores, possibly as a result of symbiosis with algae capable of consuming iron bound to the photoproduct of vibrioferrin [154]. The gene cluster responsible for the biosynthesis of vibrioferrin has been identified by transcript quantification of cultures grown in iron-limiting conditions and linked to vibrioferrin production by targeted gene disruption of the *pvsABCDE* operon [155]. Additionally, the ~7 kbp operon was also cloned from a marine metagenomics library and expressed in *E. coli* [156]. Homology searches for the genes linked to vibrioferrin biosynthesis by these methods allow for a prediction of the biosynthetic mechanism of vibrioferrin synthesis.

Vibrioferrin appears to be made from one molecule each of ethanolamine, alanine, alpha-ketoglutarate, and citric acid. It is proposed that PvsE decarboxylates free serine to produce the ethanolamine residue, which is then coupled to alanine by either PvsB or PvsD, both of which have been annotated as amide bond forming enzymes. Alpha-ketoglutaric acid is also coupled to the amine of the alanine residue by either PvsB or PvsD. PvsA then catalyzes ester formation between the terminal hydroxyl of the ethanolamine residue and citric acid. Finally, the alpha-ketoglutarate is cyclized. The pathway includes *pvsC,* which is predicted to encode a transporter allowing the secretion of vibrioferrin by the heterologous *E. coli* host. The analysis of this molecule shows the power of heterologous expression for linking genes to their molecules, but the specific function of the enzymes encoded in the *pvs* operon, as well as the mechanism for the cyclization of the 2-ketoglutarate moiety, remain untested.

## 4. Deltaproteobacteria

**Figure 6:**
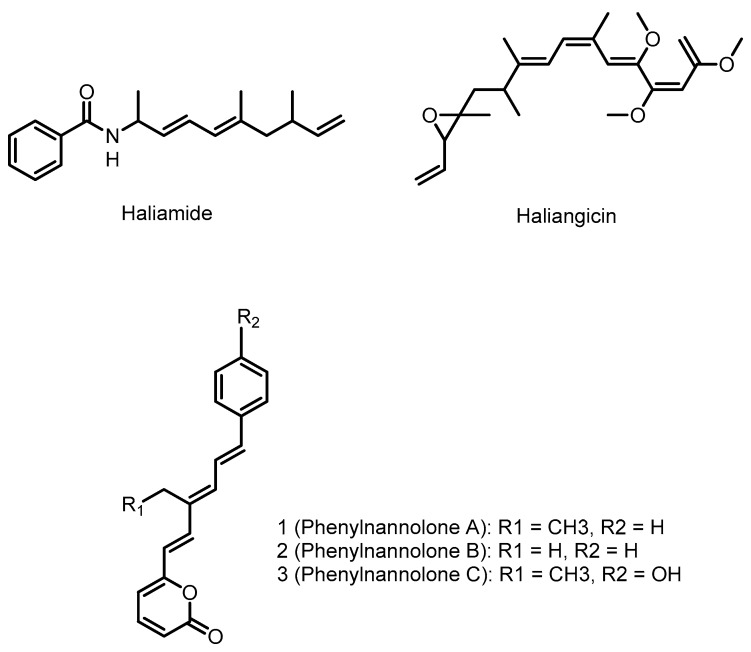
Natural products produced by marine deltaproteobacteria with known gene clusters.

The class deltaproteobacteria consists of Gram-negative aerobic as well as anaerobic genera. The aerobic genera mostly contain the fruiting body (myxospores) forming myxobacteria whereas the anaerobic deltaproteobacteria help in sulfate, sulfur and ferric iron reduction. The myxobacteria are well known for their ability to produce secondary metabolites. The characterized secondary metabolite biosynthetic pathways of deltaproteobacteria are primarily NRPS, PKS, or hybrid NRPS/PKS pathways, as seen in Figure 6. Sequencing of type I PKS genes from the myxobacteria show that they have fairly low similarity to published PKS gene sequences and are likely to represent novel PKS chemistries [157]. However, because of unfavorable factors such as slow growth rates, difficulties in isolation, strong tendency for cell aggregation and poor metabolite productivity, the discovery of new metabolites and their biosynthesis from deltaproteobacteria has been limited.

### 4.1. Haliangicin

Haliangicin is an antifungal polyketide molecule produced by the marine myxobacterium, *Haliangium ochraceum* SMP-2 [158]. Sun et al. identified the 47.8 kb *hli* biosynthetic gene cluster responsible for haliangicin biosynthesis from a cosmid library of the *H. ochraceum* genome by probing for homologues to ketosynthase genes from terrestrial myxobacteria [159]. Lambda red recombination was used to integrate the *hli* gene cluster into the genome of the fast-growing terrestrial myxobacterium, *Myxococcus xanthus,* resulting in the heterologous production of haliangicin. Isotope feeding experiments and targeted deletion of *hli* cluster genes were used to probe the role of several genes in haliangicin biosynthesis. The cluster includes genes encoding a type-I PKS (*hliFGPST*) to produce the haliangicin backbone, a β-methyl branching cassette (*hliLMNOC*), and a cassette (*hliOHIJK*) for introduction of methoxymalonate. There are also genes encoding processing enzymes such as an O-methyltransferase (*hliD*), a metallo-β-lactamase-type thioesterase (*hliE*), an acyl-CoA dehydrogenase (*hliR*), and an epoxidase (*hliU*).

**Scheme 11:**
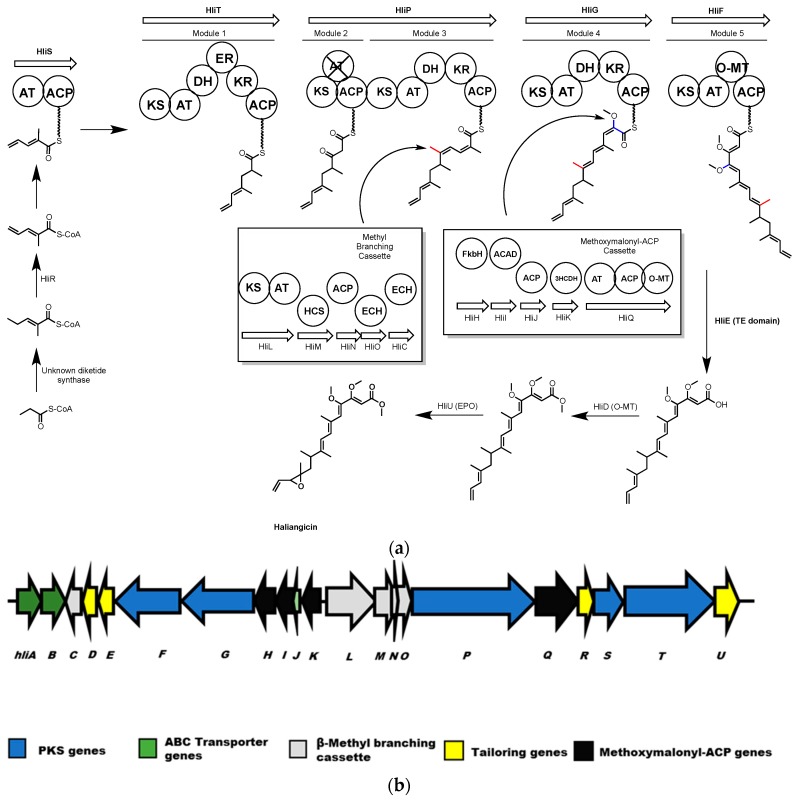
(**a**) Haliangicin biosynthesis. Polyketide synthase modules are shown as circles. Inset in this scheme are the enzymes of the methyl branching cassette and methoxymalonyl-ACP cassette. AT = acyl transferase, ACP = acyl carrier protein. KS = keto synthase, DH = dehydratase. ER = enoyl reductase, KR = ketoreductase, HCS = HMG-CoA Synthase, ACAD = acyl-CoA dehydrogenase, 3HCDH = 3-hydroxyacyl-CoA dehydrogenase, O-MT = O-methyl transferase. Non-functional domains are indicated by an X. (**b**) The *hli* gene cluster from *H. ochraceum.*

As the *hli* gene cluster has only five elongation modules, a diketide 2-methylpent-2,4-dienoyl-CoA, with an unusual terminal olefin was proposed as the starter unit for synthesizing haliangicin (Scheme 11).

This starter diketide is formed by γ,δ-dehydrogenation of 2-methylpent-2-enoyl-CoA by HliR, an acyl CoA dehydrogenase. This was confirmed in vivo by the production of 14,15-didehydrohaliangicin by an hliR mutant, and by in vitro conversion using recombinant HliR. The 2-methylpent-2,4-dienoyl CoA would be loaded onto the starter loading module of HliS, then elongated with methyl malonate and malonate, respectively, by modules 1 and 2. A β-methyl branch is then introduced at the 9-position by the methyl-branching enzymes HliL, M, N, O and C [13,160]. Module 3 incorporates another methyl malonate and then module 4 adds a glycolate extender, which is biosynthesized by the methoxymalonyl-ACP producing enzymes HliHIJKQ [161,162]. The full length polyketide is released as a carboxylic acid by HliE, which is homologous to a metallo-β-lactamase-type thioesterase [163,164], and undergoes modification by HliD and HliU to facilitate O-methylation and epoxidation respectively. HliD and HliU were each disrupted in vivo, resulting in the accumulation of several haliangicin acids in the former and 12,13-deoxyhaliangicin in the latter case.

The haliangicin gene cluster features a non-collinear gene arrangement and the PKS genes are distributed throughout the cluster, as are the gene cassettes for β-methyl branching and methoxymalonyl-ACP synthesis.

### 4.2. Phenylnannolones 

Phenylnannolones are a class of molecules produced by the marine myxobacterium *Nannocystis exedens*. These polyketide derived molecules consist of an ethyl substituted polyene flanked by a phenyl ring and a pyrone [165]. Phenylnannolone A can inhibit P-glycoprotein mediated drug efflux, a mechanism used by drug resistant tumours. Accordingly, phenylnannolone A can be used in combination with daunorubicin to improve the anti-cancer drug’s activity against resistant cells.

Feeding studies with ^13^C-labeled metabolites suggested that the molecule is derived from three and a half acetate units, one butyrate and an eight-carbon unit derived from phenylalanine [165]. The starter unit for the phenylnannolone PKS was identified as cinnamic acid based on in vitro γ-^18^O-ATP pyrophosphate exchange assays and phosphopantetheine ejection assays performed with the loading AMP ligase-PCP didomain from Phn2 [166]. Based on the feeding experiments and A-domain selectivity, the authors propose that the cinnamic acid starter unit is derived from phenylalanine, via a malonyl-CoA extended phenylacetic acid that subsequently undergoes decarboxylation. This proposed cinnamic acid biosynthesis is quite distinct from the ammonia lyase conversion of phenylalanine typically seen in bacteria. After loading of cinnamic acid the polyketide is extended with a unit of ethylmalonyl-CoA that is synthesized by Phn1, a butyryl-CoA carboxylase, rather than by a crotonyl-CoA carboxylase/reductase as is more commonly observed [167]. Subsequent addition of three acetate units in the form of malonyl-CoA gives the basic carbon backbone of phenylnannolone A. Keto-enol tautomerization at the unreduced carbonyl from module 3 is accompanied by thioesterase catalyzed cyclization to yield the pyrone moiety and phenylnannolone A. The apparent use of several uncommon or unprecedented methods to produce PKS building blocks suggest further investigations of the phenylnannolone pathway will yield valuable biosynthetic insights.

### 4.3. Haliamide

**Scheme 12:**
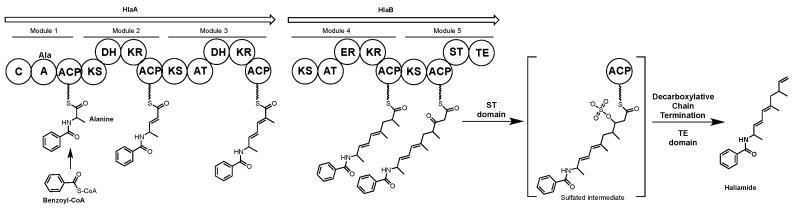
Proposed biosynthesis of haliamide. C = condensation, A = adenylation, ACP = acyl carrier protein, KS = ketosynthase, DH = dehydratase, KR = ketoreductase, AT = acyl transferase, ST = sulfotransferase.

Haliamide is a polyketide-nonribosomal peptide hybrid molecule produced by the marine myxobacterium *Haliangium ochraceum* SMP-2. Haliamide has cytotoxicity against the cervical cancer tumor cell line HeLa-S3 [168]. In vivo feeding experiments with ^13^C-labeled precursors conducted by Sun et al. showed that the biosynthetic building blocks of haliamide are benzoate, alanine, propionate and acetate [168]. The genome sequence of *H. ochraceum* SMP-2 determined by Ivanova et al. [169] was analyzed using antiSMASH [29], identifying a putative haliamide biosynthetic gene cluster (*hla*, 21.7 kbp) consisting of one gene encoding a NRPS/PKS hybrid, *hlaA*, and one gene encoding a PKS, *hlaB*, with putative sulfotransferase (ST) and thioesterase domains predicted to catalyze terminal alkene formation via decarboxylation [168]. In the proposed biosynthesis of haliamide (Scheme 12), benzoyl CoA acts as a starter unit. The *hla* gene cluster does not appear to encode a starter module for loading of benzoyl-CoA onto HlaA (or any other carrier protein), therefore the formation of the amide bond by the C domain in module 1 of HlaA may well occur between benzoyl-CoA and PCP-bound alanine, analogous to the reaction between an acyl-CoA substrate and PCP bound amino acid proposed for the biosynthesis of the macyranones [170]. The four PKS modules 2–5, incorporate two malonyl-CoAs and two methylmalonyl-CoAs to build the polyketide backbone of haliamide. The PKS modules are missing several domains, including AT domains in modules 2 and 5, a DH domain in module 3 and a KR domain in module 5. The authors propose *trans*-acting enzymes may complement the functions of the missing domains and note the presence of a stand-alone AT in the genome with predicted selectivity for malonate. During the last step of the biosynthesis, module 5 integrates an acetate unit that after hydrolysis undergoes decarboxylation to form the terminal olefin of haliamide. This last step may be mediated by the sulfotransferase and thioesterase domains found on HlaB; these domains are homologous to the *curM* ST and TE domains of the curacin A biosynthetic gene cluster from the marine cyanobacterium *Lyngbya majuscula* [171]. In curacin biosynthesis the KR domain reduces a β-keto group to the corresponding β-hydroxyl intermediate, which is then sulfated by ST to generate a good leaving group. Finally, the TE domain catalyzes hydrolysis and then decarboxylative elimination forms the terminal olefin [172]. Although no KR domain is present in module five it is possible the biosynthesis of haliamide may follow a similar termination step utilizing a *trans*-acting KR domain.

## 5. Conclusions

Marine proteobacteria offer several examples of the successful application of functional genomics towards the elucidation of biosynthetic mechanisms. While direct genetic experimentation is lacking for some of the biosynthetic gene clusters, such as those for didemnin and thalassospiramide, homology-based inquiries based on similarity to known NRPS type machinery raises interesting questions about their apparently non-canonical biosynthesis. More conventional genetic analyses have provided direct insight into the biosynthetic pathways giving rise to molecules such as tropodithietic acid and violacein, highlighting some intriguing chemistry not often emphasized in discussions relating to biosynthesis. One of the most exciting aspects of natural product research in proteobacteria is the use of heterologous expression and in vitro reconstruction of biosynthetic pathways. The common molecular biology workhorse *E. coli* is ideally suited as an expression host for many marine proteobacterial biosynthetic enzymes, due to its relatively close genetic relationship with these organisms compared with other model organisms such as *Saccharomyces cerevisiae* or *Bacillus subtilis*. This greatly facilitates both the in vivo characterization of a biosynthetic pathway, as is the case for violacein, or through the in vitro characterization of purified enzymes such as those responsible for the synthesis of pentabromopseudilin and vibriobactin. Gene clusters from metagenomic libraries are also being identified and investigated with the aid of expression in *E. coli*, many of these clusters may be from uncultured marine proteobacteria and represent both known and new biosynthetic pathways. Both the bisucaberin-producing *mbs* cluster and vibrioferrin cluster were cloned from such sources allowing greater investigation of the respective gene clusters. It is important to recognize that there are several scenarios in which it may be more challenging to produce a natural product by heterologous expression in *E. coli*, firstly if the molecule has antibacterial properties against gram-negative microbes or secondly if uncommon metabolic precursors are required for molecule biosynthesis. Control of gene expression via induction could mitigate toxicity concerns and the genetic tractability of *E. coli* as well as an abundance of bioinformatic analysis tools can allow for relatively facile metabolic engineering to generate *E. coli* strains that can produce unusual precursors (as reviewed in [173]). Proteobacteria have been used as a model for exciting new applications of genetic technology, such as the use of transformation associated recombination (TAR) to specifically clone large regions of a bacterial genome, enabling the targeted capture and heterologous expression of megasynthase gene clusters [49]. The TAR methodology has also been applied to the activation of silent clusters and when used in conjunction with the Cas9 endonuclease achieves even greater cloning efficiencies [174,175]. As the technology for targeted-cloning of large gene clusters progresses, the amount and quality of biosynthetic information related to megasynthase gene clusters will likely improve.

Enzymes used for the synthesis of natural product compounds may have considerable applications in medical and biotechnological settings. Particularly with medically important molecules such as didemnin; elucidation and engineering of the enzymatic machinery related to its synthesis may yield less toxic analogues suitable for human trials and could also serve as a means to producing large quantities of a drug molecule in an environmentally friendly manner. Identification of new scaffolds and their biosynthetic signatures in marine proteobacteria could be used to mine for related chemical structures produced in other organisms. For example, the uncommon dithiet moieties of tropodithietic acid are particularly interesting due to their rarity, and by seeking homologues of its biosynthetic genes one may identify other molecules with the same unusual structural feature. Additionally, marine proteobacterial species produce a plethora of halogenated natural products, such as the alterochromides or pentabromopseudilin [23]. Halogen substituents are prevalent amongst many medicinal and bioactive natural products and synthetic pharmaceuticals [176], so understanding how these bonds are selectively formed by living organisms could lead to the production of halogenated molecules using enzymatic semi-synthesis or metabolic engineering.

The catalogue of marine proteobacterial natural products is still much smaller than that for other families; however, the diversity of chemical structures and biosynthetic mechanisms identified thus far is encouraging. The efforts described in this review demonstrate that a wide variety of approaches have been used successfully to illuminate the specific mechanisms used by proteobacteria to synthesize molecules. Whilst following many of the same fundamental concepts as actinobacteria, we are starting to recognize proteobacterial behaviors and patterns. As sequencing technology and genome analysis tools continue to improve, the quantity of data available for a wide range of organisms will greatly increase and we must be able to connect that genomic information with chemical space. Ongoing efforts to elucidate biosynthetic approaches by marine proteobacteria, thus generating a biosynthetic language for the family, will allow the scientific community to leverage the great plethora of genetic information most effectively in our search for useful new molecules and chemical reactions.
